# Risk stratification of sudden cardiac death in asymptomatic female Brugada syndrome patients: A literature review

**DOI:** 10.1111/anec.13030

**Published:** 2023-01-11

**Authors:** Keith Sai Kit Leung, Danny Radford, Helen Huang, Ishan Lakhani, Christien Ka Hou Li, Sandeep Singh Hothi, Abraham Ka Chung Wai, Tong Liu, Gary Tse, Sharen Lee

**Affiliations:** ^1^ Cardiac Electrophysiology Unit Cardiovascular Analytics Group Hong Kong China; ^2^ Faculty of Health and Life Sciences Aston University Medical School Birmingham UK; ^3^ Kent and Medway Medical School University of Kent and Canterbury Christ Church University Canterbury UK; ^4^ University of Medicine and Health Science, Royal College of Surgeons in Ireland Dublin Ireland; ^5^ John Radcliffe Hospital, Oxford University Hospitals NHS Trust Oxford UK; ^6^ Heart and Lung Centre New Cross Hospital, Royal Wolverhampton NHS Trust Wolverhampton UK; ^7^ Emergency Medicine Unit University of Hong Kong Hong Kong China; ^8^ Tianjin Key Laboratory of Ionic‐Molecular Function of Cardiovascular Disease, Department of Cardiology Tianjin Institute of Cardiology, Second Hospital of Tianjin Medical University Tianjin China

**Keywords:** Brugada syndrome, gender difference, risk stratification, sudden cardiac death

## Abstract

**Background and Objectives:**

Risk stratification in Brugada syndrome remains a difficult problem. Given the male predominance of this disease and their elevated risks of arrhythmic events, affected females have received less attention. It is widely known that symptomatic patients are at increased risk of sudden cardiac death (SCD) than asymptomatic patients, while this might be true in the male population; recent studies have shown that this association might not be significant in females. Over the past few decades, numerous markers involving clinical symptoms, electrocardiographic (ECG) indices, and genetic tests have been explored, with several risk‐scoring models developed so far. The objective of this study is to review the current evidence of clinical and ECG markers as well as risk scores on asymptomatic females with Brugada syndrome.

**Findings:**

Gender differences in ECG markers, the yield of genetic findings, and the applicability of risk scores are highlighted.

**Conclusions:**

Various clinical, electrocardiographic, and genetic risk factors are available for assessing SCD risk amongst asymptomatic female BrS patients. However, due to the significant gender discrepancy in BrS, the SCD risk amongst females is often underestimated, and there is a lack of research on female‐specific risk factors and multiparametric risk scores. Therefore, multinational studies pooling female BrS patients are needed for the development of a gender‐specific risk stratification approach amongst asymptomatic BrS patients.

## Graphical abstract

Risk Stratification of Asymptomatic Female Brugada Syndrome Patients.

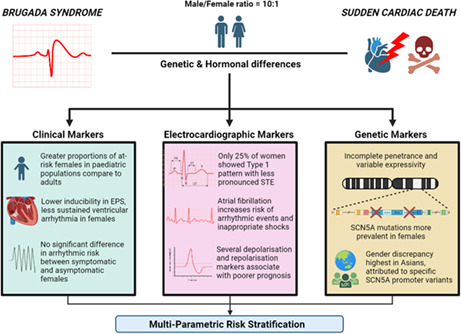

## 1 INTRODUCTION

Brugada syndrome (BrS) is the most common cardiac ion channelopathy in Asia and is characterized by coved ST‐segment elevation across the right precordial leads. (Vutthikraivit et al., 2018) Affected patients are predisposed to ventricular tachyarrhythmias, which can result in sudden cardiac death (SCD) in the absence of overt structural heart abnormalities. Currently, patients with a history of aborted SCD and/or spontaneous type 1 Brugada pattern are considered to be at the highest SCD risk. Male gender typically has a higher arrhythmic risk of over 5‐fold and a male/female ratio of around 10:1. (Milman, Gourraud, et al., 2018) In terms of features on ECG, (Sacher et al., 2008) have found that only 25% of female patients in the cohort exhibited a spontaneous type 1 pattern, of those a much less pronounced ST‐segment elevation was seen compared to human. One proposed theory suggested that hormonal difference plays a role, as humans exhibit testosterone which has been shown to increase outward repolarizing current or decrease inward depolarizing current, this ultimately accentuates ST‐segment elevation and associated risk of arrhythmic events in humans. (Liu et al., 2003; Shimizu et al., 2007) According to a recent study, initially asymptomatic patients are of comparable SCD risk to patients who were symptomatic when diagnosed with BrS. (Lee, Li, et al., 2020) Over the past decade, the need for further risk stratification among asymptomatic BrS patients has been recognized, and different risk factors have been identified. Nevertheless, most studies were typically based on male patients, while affected female received less attention, with less than 30% of literature till date highlighted gender difference. (Letsas et al., 2017; Martinez‐Barrios et al., 2022) Therefore, the aim of the present study is to summarize and evaluate the current tools in risk stratification among asymptomatic female BrS patients.

## 2 CLINICAL MARKERS

Besides clinical phenotypes, clinical characteristics have long been used as prognostic factors within the BrS cohort. Although symptomatic males are of a higher SCD risk than their asymptomatic counterparts, recent studies reported that there is no significant difference in arrhythmic risk between symptomatic and asymptomatic female patients. (Milman, Gourraud, et al., 2018; Yuan et al., 2018) Furthermore, although BrS patients typically become symptomatic during adulthood, there is a greater proportion of female patients among the pediatric cohort, suggesting that the effects of age on the SCD risk in females may differ from male patients. (Michowitz et al., 2019) Notably, a higher rate of arrhythmic events was found among the female pediatric age group. (Milman, Andorin, et al., 2018) The gender distribution of BrS patients also differs between ethnicities. It has been reported that the gender discrepancy is the highest among Asians, and the lowest among Caucasians, which may be attributed to the presence of Asian‐specific SCN5A promotor variants. (Bezzina et al., 2006) Thus, genetic polymorphism underlies the difference in risk between various gender and ethnic groups.

Although the prognostic value of ventricular tachyarrhythmia inducibility on an electrophysiological study (EPS) remains controversial (Viskin et al., 2021), affected females have been reported to encounter less sustained ventricular arrhythmias. (Rodríguez‐Mañero et al., 2021a) EPS‐positive females have been reported to be of a higher arrhythmic risk than their EPS‐negative counterparts. (Brugada et al., 2003; Kusumoto et al., 2018; Priori et al., 2012; Yuan et al., 2018) Furthermore, the extraction of the arrhythmic substrate under EPS was reported to be of prognostic value, suggesting that EPS may be useful in risk stratification regardless of sex differences. (Pappone et al., 2018) It should be noted that there were reports of lower EPS inducibility among females, though it may not equate to lower risks of spontaneous arrhythmic events. (Milman, Gourraud, et al., 2018; Rodríguez‐Mañero et al., 2021b; Sieira et al., 2016) It may be attributed to the sex difference in cardiac ion channel expression, atrioventricular node function and sinus node automaticity. (Liu et al., 2001; Tadros et al., 2014)

## 3 ELECTROCARDIOGRAPHIC MARKERS

In terms of electrocardiographic markers, spontaneous type 1 Brugada electrocardiographic pattern remains to be one of the most important. (Benito et al., 2008; Priori et al., 2012) There were reports of less frequent spontaneous type 1 pattern among female patients, though the findings are still inconsistent. (Milman, Gourraud, et al., 2018; Sieira et al., 2016; Yuan et al., 2018) The concomitant presence of non‐ventricular arrhythmias is also a predictor for ventricular tachyarrhythmia, likely due to greater instability in cardiac conduction. (Lee, Li, et al., 2020) Atrial fibrillation (AF) is one of the most common co‐existing arrhythmias. AF not only increases the risk of arrhythmic events (Kewcharoen et al., 2019), but it can also trigger inappropriate shocks in patients with implantable cardioverter‐defibrillators in addition to increasing patients' stroke risk, thus impairs the quality of life of those affected. (Tse, Lee, Mok, et al., 2020; Veltmann et al., 2010)

The prognostic value of repolarization and depolarization electrocardiographic indices has been explored. In terms of depolarization‐related risk factors, QRS‐interval fragmentation and prolongation, epsilon‐like waves, large S wave of at least 40 ms, and the presence of late‐potentials were poor prognostic markers reported. (Asvestas et al., 2018) These markers support the depolarization hypothesis, which proposes that the reduced conduction velocity of the propagating action potential underlies the arrhythmic substrate. (Tse et al., 2016) By contrast, repolarization‐related risk factors include the occurrence of early repolarization pattern, QTc prolongation, T_peak_‐T_end_ interval prolongation, and its dispersion in addition to T‐wave alternans supports the repolarization hypothesis (Asvestas et al., 2018; Tse et al., 2018). Besides standalone electrocardiographic markers, the prognostic value of electrocardiographic temporal variability has been explored. A reason study demonstrated that increased temporal variability in the aforementioned electrocardiographic markers, in addition to novel markers such as ST‐elevation in lead V3, is predictive of SCD. (Lee, Zhou, et al., 2020) The involvement of temporal variability accounts for the dynamicity in the electrical conduction of BrS patients, thus providing additional benefits in risk stratification. However, the diffeent published studies have not evaluated the predictive values of these ECG markers specifically for the female subpopulation.

## 4 GENETIC MARKERS

Genetic mutations have been used as a prognostic tool for BrS patients. Studies have shown that BrS demonstrate incomplete penetrance and variable expressivity. (Poelzing et al., 2006; Wijeyeratne et al., 2020) Mutations in the SCN5A gene, which codes for the alpha subunit of the sodium channel, are most commonly found, reported in around 20% of patients. (Kapplinger et al., 2010; Probst et al., 2010) The mutation was reported as an SCD predictor by the Survey on Arrhythmic Events in BRUgada Syndrome (SABRUS) registry and was found to be more prevalent among females. (Milman, Gourraud, et al., 2018; Yamagata et al., 2017) Mutations at different sites of the gene result in varying extents of sodium channel functional loss, consequentially resulting in individual variations in the clinical presentation and SCD risk between different patients. (Meregalli et al., 2009; Tse, Lee, Liu, et al., 2020) BrS was also found to be associated with mutations in genes coding for other ion channels, including potassium, calcium, and hyperpolarization‐activated cyclic nucleotide gated‐channels. (Roden, 2010)

## 5 MULTI‐PARAMETRIC RISK‐STRATIFICATION

Given the multi‐factorial nature underlying the increased SCD risk among BrS patients, a multi‐parametric approach that integrates clinical, electrocardiographic, and genetic factors is warranted for risk stratification. In the past ten years, several risk scores have been developed by different groups with some predictive power. (Chung et al., 2022; Delise et al., 2011; Honarbakhsh et al., 2021; Kawada et al., 2018; Kawazoe et al., 2016; Lee et al., 2022; Okamura et al., 2015; Sieira et al., 2017) However, their inability to account for the latent relationships between risk factors limited their prognostic value, thus restricts their clinical application. The recent introduction of machine‐learning techniques to the multi‐parametric risk scores helps to resolve this problem. For example, a recent study used the non‐negative matrix factorization model to account for the inter‐variable relationships between clinical and electrocardiographic indices, which demonstrated better predictive performance than simply using logistic regression. (Tse, Zhou, Lee, et al., 2020) The current risk scores are based on male‐predominant cohorts, hence did not account for the difference in disease manifestation and pathogenic mechanisms in females. Thus, further research on multi‐parametric risk scores based on female cohorts is warranted, and modification of current risk score models that take gender and genetic polymorphisms into consideration is crucial.

## 6 CONCLUSION

In conclusion, there are a plethora of clinical, electrocardiographic, and genetic risk factors underlying the SCD risk among asymptomatic female BrS patients. However, due to the significant gender discrepancy in BrS, the SCD risk among females is often underestimated, and there is a lack of research on female‐specific risk factors and multi‐parametric risk scores. Therefore, multi‐national studies pooling female BrS patients are needed for the development of a gender‐specific risk stratification approach among asymptomatic BrS patients.

## AUTHOR CONTRIBUTIONS

KSKL carried out study conception, project planning, manuscript drafting, and critical review of the manuscript. GT and SL carried out study conception, project planning, and critical review of the manuscript. All remaining authors (DR, HH, IL, CKHL, SH, AKCW, TL) carried out critical review of the manuscript.

## FUNDING INFORMATION

No funding was received.

## CONFLICT OF INTEREST

The authors declare that they have no competing interests.

## CONSENT FOR PUBLICATION

All authors consent to publication.

## ETHICS STATEMENT

Not applicable.

## Data Availability

Data sharing is not applicable to this article as no new data were created or analyzed in this study.
